# Early antiviral treatment in outpatients with COVID-19 (FLARE): a structured summary of a study protocol for a randomised controlled trial

**DOI:** 10.1186/s13063-021-05139-2

**Published:** 2021-03-08

**Authors:** Li-An K. Brown, Nick Freemantle, Judy Breuer, Hakim-Moulay Dehbi, Kashfia Chowdhury, Gemma Jones, Felicia Ikeji, Amalia Ndoutoumou, Krishneya Santhirakumar, Nicky Longley, Anna M. Checkley, Joseph F. Standing, David M. Lowe

**Affiliations:** 1 UCL Institute of Immunity and Transplantation, Royal Free Hospital, Pond Street, London, NW3 2QG UK; 2 Comprehensive Clinical Trials Unit, University College London, 90 High Holborn, 2nd Floor, London, WC1V 6LJ UK; 3 Division of Infection and Immunity, University College London, London, UK; 4 Hospital for Tropical Diseases, University College London Hospitals NHS Foundation Trust, London, UK; 5 London School of Hygiene and Tropical Medicine, London, UK; 6 Infection, Inflammation, Immunity Section, UCL Great Ormond Street Institute of Child Health, University College London, 30 Guilford Street, London, WC1N 1EH UK

**Keywords:** COVID-19, randomised controlled trial, protocol, factorial design, placebo-controlled trial, favipiravir, lopinavir/ritonavir, antivirals, combination therapy, early treatment

## Abstract

**Objectives:**

The objective of this trial is to assess whether early antiviral therapy in outpatients with COVID-19 with either favipiravir plus lopinavir/ritonavir, lopinavir/ritonavir alone, or favipiravir alone, is associated with a decrease in viral load of SARS-CoV-2 compared with placebo.

**Trial design:**

FLARE is a phase IIA randomised, double-blind, 2x2 factorial placebo-controlled, interventional trial.

**Participants:**

This trial is being conducted in the United Kingdom, with Royal Free Hospital, London as the lead site. Participants are non-hospitalised adults with highly suspected COVID-19 within the first 5 days of symptom onset, or who have tested positive with SARS-CoV-2 causing COVID-19 within the first 7 days of symptom onset, or who are asymptomatic but tested positive for SARS-CoV-2 for the first time within the last 48 hours.

Inclusion criteria are as follows:

1. Any adult with the following:

- Symptoms compatible with COVID-19 disease (Fever >37.8°C on at least one occasion AND either cough and/ or anosmia) within the first 5 days of symptom onset (date/time of enrolment must be within the first 5 days of symptom onset)
- OR ANY symptoms compatible with COVID-19 disease (may include, but are not limited to fever, cough, shortness of breath, malaise, myalgia, headache, coryza) and tested positive for SARS-CoV-2 within the first 7 days of symptom onset) (date/time of enrolment must be within the first 7 days of symptom onset)
- OR no symptoms but tested positive for SARS-CoV-2 within the last 48 hours (date/time of test must be within 48 hours of enrolment)

2. Male or female aged 18 years to 70 years old inclusive at screening
3. Willing and able to take daily saliva samples
4. Able to provide full informed consent and willing to comply with trial-related procedures

Exclusion criteria are as follows:

1. Known hypersensitivity to any of the active ingredients or excipients in favipiravir and matched placebo, and in lopinavir/ritonavir and matched placebo (See Appendix 2)
2. Chronic liver disease at screening (known cirrhosis of any aetiology, chronic hepatitis (e.g. autoimmune, viral, steatohepatitis), cholangitis or any known elevation of liver aminotransferases with AST or ALT > 3 X ULN)*
3. Chronic kidney disease (stage 3 or beyond) at screening: eGFR < 60 ml/min/1.73m^2^ *
4. HIV infection, if untreated, detectable viral load or on protease inhibitor therapy
5. Any clinical condition which the investigator considers would make the participant unsuitable for the trial
6. Concomitant medications known to interact with favipiravir and matched placebo, and with lopinavir/ritonavir and matched placebo, and carry risk of toxicity for the participant
7. Current severe illness requiring hospitalisation
8. Pregnancy and/ or breastfeeding
9. Eligible female participants of childbearing potential and male participants with a partner of childbearing potential not willing to use highly effective contraceptive measures during the trial and within the time point specified following last trial treatment dose.
10. Participants enrolled in any other interventional drug or vaccine trial (co-enrolment in observational studies is acceptable)
11. Participants who have received the COVID-19 vaccine

*Considering the importance of early treatment of COVID-19 to impact viral load, the absence of known chronic liver/ kidney disease will be confirmed verbally by the participant during pre-screening and Screening/Baseline visit. Safety blood samples will be collected at Screening/Baseline visit (Day 1) and test results will be examined as soon as they become available and within 24 hours.

**Intervention and comparator:**

Participants will be randomised 1:1:1:1 using a concealed online minimisation process into one of the following four arms:

- Arm 1: Favipiravir + Lopinavir/ritonavir

Oral favipiravir at 1800mg twice daily on Day 1, followed by 400mg four (4) times daily from Day 2 to Day 7 PLUS lopinavir/ritonavir at 400mg/100mg twice daily on Day 1, followed by 200mg/50mg four (4) times daily from Day 2 to Day 7.

- Arm 2: Favipiravir + Lopinavir/ritonavir placebo

Oral favipiravir at 1800mg twice daily on Day 1, followed by 400mg four (4) times daily from Day 2 to Day 7 PLUS lopinavir/ritonavir matched placebo at 400mg/100mg twice daily on Day 1, followed by 200mg/50mg four (4) times daily from Day 2 to Day 7.

- Arm 3: Favipiravir placebo + Lopinavir/ritonavir

Oral favipiravir matched placebo at 1800mg twice daily on Day 1, followed by 400mg four (4) times daily from Day 2 to Day 7 PLUS lopinavir/ritonavir at 400mg/100mg twice daily on Day 1, followed by 200mg/50mg four (4) times daily from Day 2 to Day 7.

- Arm 4: Favipiravir placebo + Lopinavir/ritonavir placebo

Oral favipiravir matched placebo at 1800mg twice daily on Day 1, followed by 400mg four (4) times daily from Day 2 to Day 7 PLUS lopinavir/ritonavir matched placebo at 400mg/100mg twice daily on Day 1, followed by 200mg/50mg four (4) times daily from Day 2 to Day 7.

**Main outcomes:**

The primary outcome is upper respiratory tract viral load at Day 5.

Secondary outcomes:

- Percentage of participants with undetectable upper respiratory tract viral load after 5 days of therapy
- Proportion of participants with undetectable stool viral load after 7 days of therapy
- Rate of decrease in upper respiratory tract viral load during 7 days of therapy
- Duration of fever following commencement of trial medications
- Proportion of participants with hepatotoxicity after 7 days of therapy
- Proportion of participants with other medication-related toxicity after 7 days of therapy and 14 days post-randomisation
- Proportion of participants admitted to hospital with COVID-19 related illness
- Proportion of participants admitted to ICU with COVID-19 related illness
- Proportion of participants who have died with COVID-19 related illness
- Pharmacokinetic and pharmacodynamic analysis of favipiravir
- Exploratory: Proportion of participants with deleterious or resistance-conferring mutations in SARS-CoV-2

**Randomisation:**

Participants will be randomised 1:1:1:1 using a concealed online minimisation process, with the following factors: trial site, age (≤ 55 vs > 55 years old), gender, obesity (BMI <30 vs ≥30), symptomatic or asymptomatic, current smoking status (Yes = current smoker, No = ex-smoker, never smoker), ethnicity (Caucasian, other) and presence or absence of comorbidity (defined as diabetes, hypertension, ischaemic heart disease (including previous myocardial infarction), other heart disease (arrhythmia and valvular heart disease), asthma, COPD, other chronic respiratory disease).

**Blinding (masking):**

Participants and investigators will both be blinded to treatment allocation (double-blind).

**Numbers to be randomised (sample size):**

240 participants, 60 in each arm.

**Trial Status:**

Protocol version 4.0 dated 7^th^ January 2021. Date of first enrolment: October 2020. Recruitment is ongoing, with anticipated finish date of 31^st^ March 2021.

**Trial registration:**

The FLARE trial is registered with Clinicaltrials.gov, trial identifying number NCT04499677, date of registration 4^th^ August 2020.

**Full protocol:**

The full protocol is attached as an additional file, accessible from the Trials website (Additional file 1). In the interest in expediting dissemination of this material, the familiar formatting has been eliminated; this Letter serves as a summary of the key elements of the full protocol.

**Supplementary Information:**

The online version contains supplementary material available at 10.1186/s13063-021-05139-2.

## Supplementary Information

**Additional file 1.** Full study protocol.

## Data Availability

The Chief Investigator, Clinical Project Manager, Trial Manager, Data Manager, Statisticians and trial management team have full access to the trial data. Requests for access to trial data will be considered, and approved in writing where appropriate, after formal application to the Trial Steering Committee. Considerations for approving access are documented in the Trial Steering Committee Terms of Reference.

